# Facilitator competency rubric in nursing simulations: transcultural adaptation and validation of the German version

**DOI:** 10.1186/s12912-023-01317-6

**Published:** 2023-04-26

**Authors:** Theresa A. Forbrig, Paul Gellert, Maria Biniok, Johannes Gräske

**Affiliations:** 1 Department II - Health, Education and Pedagogy, Alice Salomon Hochschule Berlin University of Applied Science, Alice-Salomon-Platz 5, 12627 Berlin, Germany; 2 Institute of Medical Sociology and Rehabilitation Science, Charité – Universitätsmedizin Berlin, Berlin, Germany

**Keywords:** Facilitator Competency Rubric, Simulation pedagogy, Facilitator’s competence, Self-assessment tool, Nursing

## Abstract

**Background:**

Simulations are part of nursing education. To obtain good results, simulation facilitators need to be competent in simulation pedagogy. Part of this study was the transcultural adaptation and validation of the Facilitator Competency Rubric into German (FCR_G_) and the evaluation of the factors associated with higher competencies.

**Method:**

A written-standardized cross-sectional survey was conducted. *N* = 100 facilitators (mean age: 41.0 (9.8), female: 75.3%) participated. Test–re-test, confirmatory factor analysis (CFA), and ANOVAs were conducted to evaluate the reliability and validity of, and the factors associated with, FCR_G_. Intraclass correlation coefficient (ICC) values > .9 indicate excellent reliability.

**Results:**

The FCR_G_ achieved good intra-rater reliability (all ICC > .934). A moderate correlation (Spearman-rho .335, *p* < .001) with motivation indicates convergent validity. The CFA showed sufficient to good model fits (CFI = .983 and SRMR = .016). Basic simulation pedagogy training is associated with higher competencies (*p* = .036, b = 17.766).

**Conclusion:**

The FCR_G_ is a suitable self-assessment tool for evaluating a facilitator’s competence in nursing simulation.

## Introduction

Although simulations in nursing education have been established for a long time, [1] they have only just started in Germany, because of a new nursing Act. This Act explicitly allows nursing simulation hours to be counted as practical hours, which will give a boost to nursing simulations and will require them to be adapted to international standards within the next few years [2]. Simulation is defined as “an educational strategy in which a particular set of conditions are created or replicated to resemble authentic situations that are possible in real life. Simulation can incorporate one or more modalities to promote, improve, or validate a participant’s performance” [3]. Simulation can increase patients’ safety and nurses’ confidence, reduce errors in patient care, and teach nursing skills and competencies. Another important effect is to increase learners’ confidence in preparing for clinical practice [4]. More and more US nursing administrations follow the National Council of State Boards of Nursing (NCSBN) statement that up to 50% of real-life practical hours could be substituted by simulations. This development is internationally desirable. For this substitution, competent facilitators are a prerequisite [5].

## Background

Simulations in nursing education are guided by at least one facilitator. Facilitators define the conceptual context, including the alignment and distribution of the learning objectives. Additionally, they evaluate whether students are prepared with the necessary knowledge, skills, and abilities to take part in certain simulations [6]. Facilitators ensure that students are well prepared and have all the necessary information for the upcoming simulation. They instruct students, in order to obtain a good balance of challenge and burden [7]. During the simulation, facilitators focus on the simulation in order to adapt it to achieve the learning objectives. Johnston et al. (2018) [8] highlighted the ability to reflect [8]. Facilitators are able to apply systematized debriefing strategies and give feedback with respect to the learning situation of the participants. Finally, facilitators should evaluate themselves and their simulation in order to develop or redesign the curricula. There is a need for an external perspective to ensure the continuous qualitative improvement of facilitators [9].

### Competencies

A facilitator should have basic training in simulation pedagogy through formal coursework, and should participate in ongoing advanced training [10]. Competency can be defined as the ability of an individual to perform adequately in a given context [11]. An experienced mentor should evaluate the facilitator’s competency at least once per semester, and this evaluation should be flanked by the facilitator performing self-assessments of their own competency [12]. A higher motivation to facilitate simulations in nursing is associated with higher competencies [13].

### Assessment of facilitator competencies

Current recommendations for competency development in simulation teaching emphasize an orientation towards the standards of the International Nursing Association for Clinical Simulation and Learning (INACSL) [14, 15]. The Facilitator Competency Rubric (FCR) was developed on the basis of the novice-to-expert theory of Benner (1984), with respect to the INACSL standards, and includes five domains (see Table 1) [16]. The FCR is an up-to-date international assessment tool that addresses the competencies defined by the INACSL. The aim of the present study was to translate the FCR into German and to psychometrically test this version as a self-assessment instrument for facilitator competencies. Furthermore, factors associated with higher competencies were evaluated. The use and dissemination of established, evidence-based questionnaires is desirable. The overall aim is an internationally comparable competence measurement of simulation facilitators. This will allow an exchange of the experiences and knowledge of simulation facilitators between different countries.

**Table 1:** FCR domains

Domain	Number of items	Addresses...
Preparation	7	...the importance of defining learning objectives
Prebriefing	4	...confidence, code of conduct, participation, and respect
Facilitation	6	...setting focus, change of instructions, engagement of participants, performance and time management
Debriefing	8	...facilitation of reflection, extent to which all facilitators and participants discuss, debate, or analyze simulation activities
Evaluation	4	...the willingness of facilitators to change and adapt upcoming simulations

## Methods

The survey took place as part of the SkilsLab:XR project (4/2020 - 10/2022). The primary goal of the project was to evaluate XR technologies in the context of simulation-based nursing education.

In the present sub-study, the FCR instrument was translated and psychometrically tested. A written, standardized, cross-sectional survey was conducted between November 2021 and February 2022. Data were collected using the online questionnaire QUAMP®.

### Sample

The study focused on universities, vocational schools, and advanced training institutions in Germany. To avoid selection bias, a comprehensive recruiting strategy was applied. The link to the survey was sent by e-mail to members of the Deans’Conference of Nursing Science, an association of universities running nursing programmes (*n* = 62), to schools on a register of vocational schools (*n* = 1,185), and to further education centres and established simulation networks (*e.g.,* SimNat^®^) in nursing (*n* = 3). A reminder was sent after six weeks. Key persons were asked to forward the e-mail to facilitators. The e-mail included a covering letter containing information about the study and the data protection concept. Facilitators had to agree online to participate before they could start the questionnaire. Those included were facilitators in nursing simulation in universities, vocational schools, and advanced training institutions, who spoke German and who agreed to participate.

### Instrument

The first part of the questionnaire includes typical characteristics of the participants (e.g., age and sex), but also information about their education in the field of simulation science (e.g. ‘Have you had basic training in simulation pedagogy?’). The respondents’ motivation to engage in simulation facilitation was assessed by a self-rated 3-point Likert scale (no, medium to high, very high). In Germany, up to 2020, nursing education was separated into adult, paediatric, and elderly care. Participants were asked about working in these three fields. The whole questionnaire was self-administered.

### Facilitator Competency Rubric (FCR)

Originally, the FCR was developed as an observational tool, and it was first published in 2018 [16]. However, the authors suggested that the instrument could be used as a self-rating instrument [16]. The FCR_G_ was therefore applied as a self-administered version. The FCR_G_ questionnaire includes 29 questions on five domains (see Table 1). For each question, a self-assessment of competencies is made on a 5-point Likert scale, which yields three categories (beginners to advanced beginners; competent; proficient to expert). The evaluation is performed by adding up the respective answers. For the total scale, the theoretical range is 29-145. Higher scores indicate higher competencies. The original version was found to have good psychometric properties (Goodman-Kruskall-Gamma = .84) [16].

### Translation and cultural adaptation

Permission to translate the FCR into German was obtained from Dr Kim Leighton. The translation process followed the recommendations of Beaton et al. for self-ratings [16]. A five-step procedure was conducted. Step 1 Translation: Two independent forward translations into German were performed. Step 2 Synthesis: A working group discussed the differences until a consensus was found. Step 3 Back translation: A “blind to the original version” back translation was carried out as recommended, as a validity check. Step 4 Approval by original author: The author of the original version received the back translation and gave their clearance. Step 5 Pre-testing: The German version of the FCR was pre-tested by four facilitators (who were not part of the working group) to identify difficulties with items or responses.

### Statistical analyses

Means and standard deviations were used to describe the data. In order to evaluate the psychometric properties, reliability and validity were analysed. Group differences were analysed using ANOVA and Chi-square tests. The data analysis was done using SPSS version 28 [17].

#### Reliability

Internal consistency was measured using Cronbach’s alpha. If all items measure the same latent variable, Cronbach’s alpha tells us how well these items measure this latent variable. A value greater than .7 indicates sufficient internal consistency [18]. In addition, test–re-test reliability was assessed using the intraclass correlation coefficient (ICC). A pragmatic subsample completed the questionnaire twice, within two weeks on average. ICC values of greater than .9 indicate excellent reliability [19].

#### Validity

Confirmatory factor analysis (CFA) was performed to assess the structure of the FCR_G_. CFA was performed using R 4.2.0 (lavaan package) [20, 21]. As recommended by Hu and Bentler (2009), the comparative fit index (CFI) and standardized root mean square residual (SRMR) were calculated. For small sample sizes (*n* < 250), they recommend CFI values of greater than .95 and SRMR of less than .06 [22]. For the convergent validity estimation, the two-sided Pearson’s correlation with motivation was examined.

Because of the small sample size and the rather complex structure of the FCR, item parcelling was used to reduce the number of manifest parameters and to define the structure of the model. In addition, this procedure increases the stability of the parameter estimates [23]. A package is used to represent the average of several items, and this is considered an indicator of the latent construct. Each item was assigned to a package according to the content of the package and the item. Each facet of the subdomain was assigned to a parcel. Two manifest indicators for each latent construct were calculated.

In order to explain the FCR_G_ scores, ANOVA models were used. The dependent variables were the five subdomains and the total score for the FCR_G_. The independent variables were: sex, area of work (adult, paediatric, or elderly care), area (academic, vocational, or advanced training), basic training (yes/no), current direct care (yes/no), working experience in nursing (years), and years of simulation facilitation. For all the statistical analyses, the model assumptions were tested. Analyses were performed at the significance level of less than or equal to .05.

## Results

### Translation and cultural adaptation

**Fig. 1:**
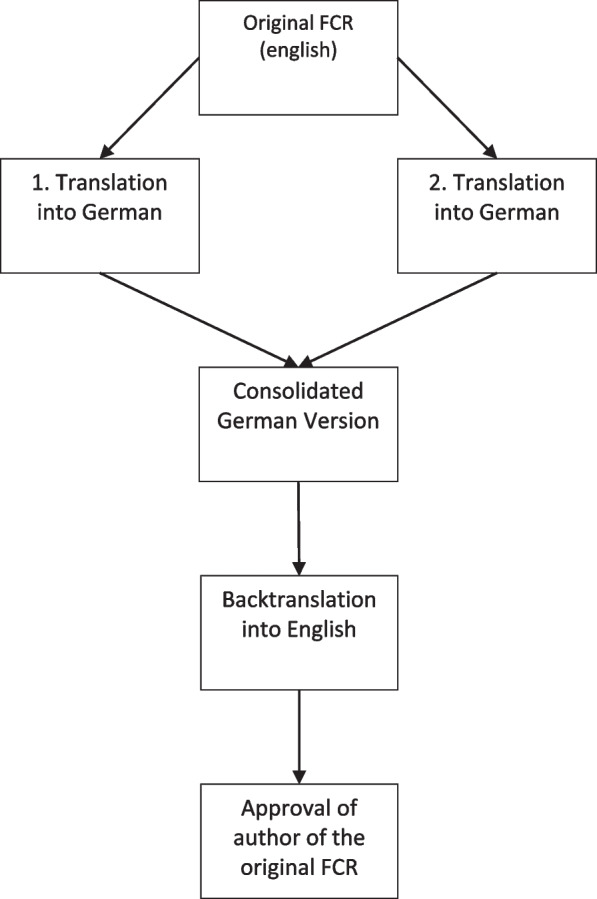
Translation process

The procedure for the translation and the cultural adaptation is described in the Method section. The five-step process yielded an agreed German version of the FCR (see Fig. 1).

#### Step 1: translation

The initial forward translations were, in general, similar. Differences were identified in the “Preparation” and “Debriefing” Items. In the “Preparation” item and the related subcategories (“Learning Objectives”, “Fidelity Level” and “Evaluation Methods”), the discrepancies were discussed. In addition, in the “Debriefing” category, linguistic smoothing of nonconformities with respect to the “Model/Plan” item was performed in a discussion.

#### Step 2: synthesis

The German versions were compared independently by the authors. There were only minor linguistic differences. One version was agreed upon. The differences that existed were discussed with four further facilitators with different levels of experience, so that a consolidated version was created. This means that the four other facilitators had an advisory and testing role. The result was a consolidated German version of the FCR (FCR_G_).

#### Step 3: back translation

The merged German version was translated back into English by a professional native English-speaking translator. This translator was blinded regarding the original version.

#### Step 4: expert committee review

The back-translated version was critically reviewed by the present authors. No differences between the back translation and the original version were found. The back translation was submitted to Dr Kim Leighton (the developer of the original version) for review, in case there were differences in the conceptual content (see Fig. 1). Clearance was obtained from Dr Leighton.

#### Step 5: pre-testing

Before the validation process started, pre-testing was conducted with four facilitators. No difficulties with the items were identified.

The present study involved 100 simulation facilitators. Because of the recruitment strategy used, a response rate cannot be calculated. The participants were from universities, vocational schools, and advanced training facilities in German nursing education. Of them, 75.3% were female and 24.7% were male; no participant was diverse. The mean age of the participants was 41.2 (9.8) years (see Table 2). Most of the participants were engaged in adult care (according to the German separation of the nursing profession) across all the nursing sectors. About one quarter of the participants held a bachelor’s degree as their highest qualification, and more than half held a master’s degree. The average length of the participants’ practical work experience was 14.7 (9.9) years. Their last activity in direct patient care was, on average, 6.9 (5.5) years before the test was taken. No significant differences between the institutions were found for any sample characteristics (all *p* > .05). However, there were significantly fewer people with basic training in vocational schools compared to the other areas (Chi-square *p* = .002).

**Table 2:** Sample characteristics (*n* = 100)

	Total (*n* = 100)	University (*n* = 23)	Vocational school (*n* = 63)		Advanced training institution (*n* = 8)	Group comparison
**Age** in years, mean (sd)						*p* = .484
	41.0 (9.5)	40.8 (10.2)	40.5 (8.9)		44.9 (12.3)	
**Sex**, % (n)						*p* = .610
Female	75.3 (70)	72.7 (16)	77.8 (49)		62.5 (5)	
Male	24.7 (23)	27.3 (6)	22.2 (14)		37.5 (3)	
**Nursing profession,** % (n)						*p* = .673
Adult	79.0 (64)	78.9 (15)	76.8 (43)		100 (6)	
Pediatric	12.3 (10)	15.8 (3)	12.5 (7)		0 (0)	
Elderly care	8.6 (7)	5.3 (1)	10.7 (6)		0 (0)	
**Highest academic degree**						*p* = .060
Bachelor	48.9 (46)	30.4 (7)	52.4 (33)		75.0 (6)	
Master or comparable	51.1 (48)	69.6 (16)	47.6 (30)		25.0 (2)	
**Work experience nursing,** mean (sd)						*p* = .059
Years	14.7 (9.9)	13.3 (10.2)	14.3 (9.2)		23.1 (12.4)	
**Time since last work in nursing,** mean (sd)						*p* = .083
Years	6.9 (5.5)	5.8 (3.9)	7.0 (5.8)		15.0 (7.1)	
**Basic training in simulation pedagogy** % (n)						*p* = .002
Yes	17.0 (16)	30.4 (7)	7.9 (5)		50.0 (4)	
**Years of simulation facilitation,** mean (sd)						*p* = .802
Years	5.0 (6.3)	5.2 (6.0)	4.8 (6.4)		6.4 (6.9)	
**Motivation for simulation facilitation**, % (n)						n/a
Low	4.3 (4)	4.3 (1)	4.8 (3)		0.0 (0)	
Medium high	45.7 (43)	30.4 (7)	52.4 (33)		37.5 (3)	
Very high	50.0 (47)	65.2 (15)	42.9 (27)		62.5 (5)	

*sd* standard deviation, *n/a* not applicable; Group comparison for continuous variables: ANOVA; for categorial variables: chi-square-test

### Competencies of the simulation facilitators

On average, the facilitators had a score of 89.4 (25.6) on the FCR_G_. The median competence level on each subdomain was “competent”. A low proportion of the facilitators evaluated themselves as being “proficient to expert”. In the preparation domain, the lowest number of facilitators (*n* = 8) was at the “proficient to expert” level (see Table 3).

**Table 3:** Competence regarding FRC_G_

	Total (29-145)	Preparation (7-35)	Prebriefing (4-20)	Facilitation (6-30)	Debriefing (8-40)	Evaluation (4-20)
**FCR**_**G**_** score**, mean (sd)	89.4 (25.6)	20.3 (5.9)	11.7 (4.1)	19.6 (5.4)	25.3 (7.8)	12.5 (3.9)
Min-max	29-140	7-33	4-19	6-30	8-40	9-20
**FCR**_**G**_ category, % (n)						
Beginner-advanced beginner	n/a	16.0 (16)	18.0 (18)	12.0 (12)	14.0 (24)	15.0 (15)
Competent	n/a	76.0 (76)	62.0 (62)	66.0 (66)	64.0 (64)	64.0 (64)
Skilled to expert	n/a	8.0 (8)	20.0 (20)	22.0 (22)	21.0 (21)	21.0 (21)

Scoring categories: beginner to advanced, competent, skilled to expert

Preparation: 7-14, 15-27, 28-35; prebriefing: 4-8, 9-15, 16-20; facilitation: 6-12, 13-23, 24-30; debriefing: 8-16, 17-31, 32-40; evaluation: 4-8, 9-15, 16-20

### Reliability

All of the domains achieved sufficient internal consistency, as they reached values above .7 (see Table 4). The ICC of all the domains indicates an excellent intra-rater reliability (all > .9).

**Table 4:** Reliability of the FCR_G_

	Total (*n* = 100)	Preparation (*n* = 100)	Prebriefing (*n* = 100)	Facilitation (*n* = 100)	Debriefing (*n* = 100)	Evaluation (*n* = 100)
Cronbach's alpha	.980	.867	.909	.942	.960	.889
ICC, *p* value	.986 *p* < .001	.934 *p* = .004	.938 *p* = .006	.994 *p* < .001	.982 *p* < .001	.993 *p* < .001

### Validity

**Fig. 2:**
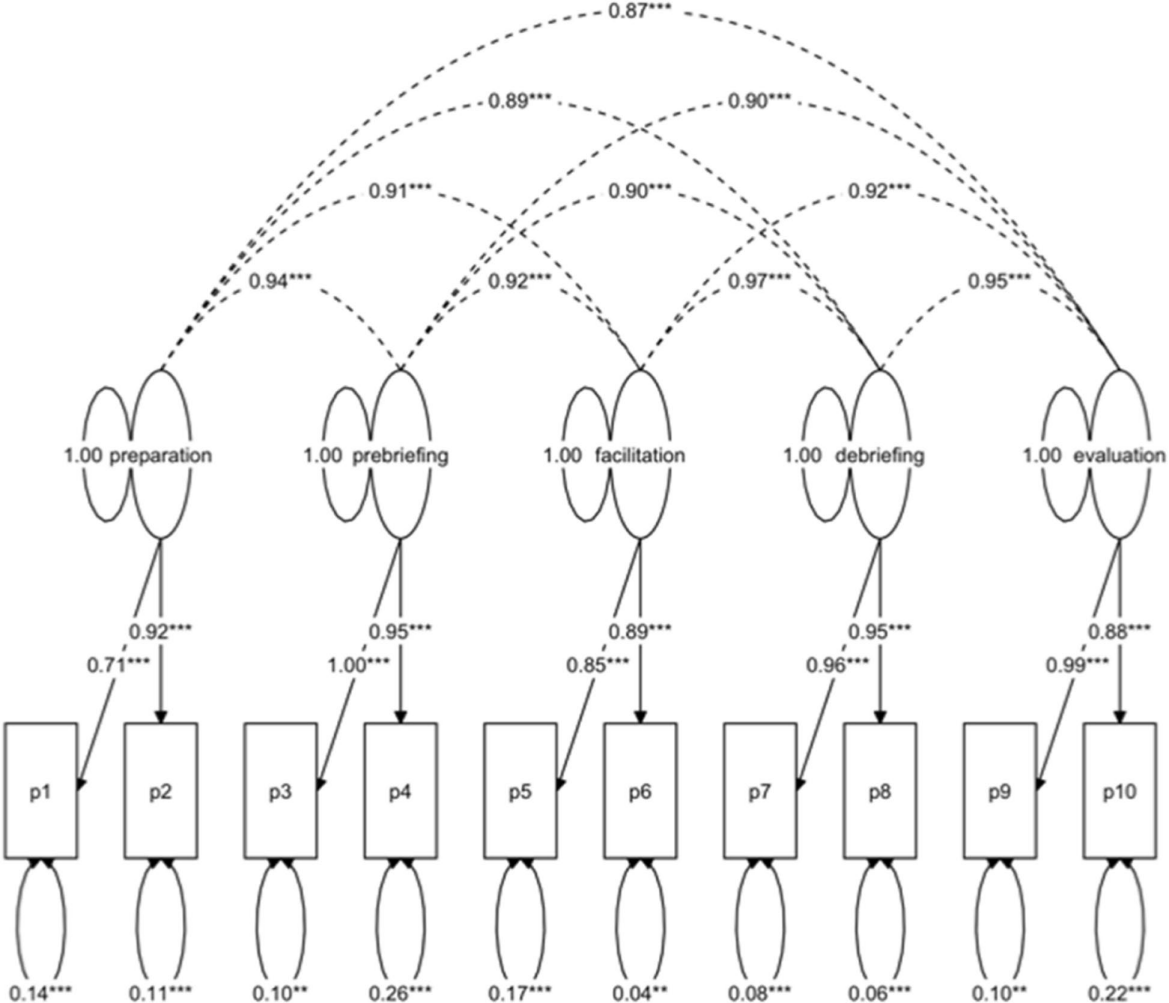
structure model of FCR_G_

There is a significant moderate positive correlation (Spearman-rho .335, *p* < .001) between motivation and simulation facilitator competencies, indicating convergent validity. The CFA of the model (see Fig. 2) shows sufficient to good model fits, with CFI of .983 and SRMR of .016, which are values in the acceptable range.

### Factors associated with higher competencies

The factors associated with higher facilitation competencies in simulation are shown in Table 5. The results show that the independent variables sex, profession and area have no influence on self-rated competencies regarding FCR_G_. The independent variable basic training is significantly associated with competencies for the total score, the preparation domain, and the debriefing domain. Continuing to work in direct care is negatively associated with scores for the facilitation domain and the debriefing domain. The maximum proportion of explained variance is corr. R^2^ = .149.

**Table 5:** ANOVA - FCR_G_

Independent variable	Total		Preparation		Prebriefing		Facilitation		Debriefing		Evaluation	
	b	*p*-value	b	*p*-value	b	*p*-value	b	*p*-value	b	*p*-value	b	*p*-value
Corr. model		**.**054		**.032**		.128		.052		**.023**		.469
Sex^a^												
Female	-4.288	.564	-1.485	.387	-1.022	.414	-.873	.582	-.718	.738	-.170	.887
Profession^b^												
Adult	2.399	.839	1.654	.544	-.199	.920	-.225	.929	1.297	.704	-.552	.772
Pediatric	5.138	.713	3.355	.302	1.298	.583	-.990	.741	1.129	.780	.353	.876
Area^c^												
University	21.458	.101	5.845	.055	3.940	.075	4.660	.097	4.294	.254	2.701	.200
Vocational school	8.375	.504	2.533	.384	2.072	.329	2.202	.413	-.641	.860	.925	.649
Basic training^d^												
Yes	**17.766**	**.036**	**4.118**	**.035**	2.321	.101	3.215	.074	**6.507**	**.008**	1.593	.238
Direct care^e^												
Yes	-13.697	.131	-1.979	.343	-1.419	.351	**-4.287**	**.029**	**-5.242**	**.047**	-.785	.590
Years of work experience nursing^f^	.114	.656	.032	.665	.035	.516	.032	.639	.066	.475	-.024	.646
Years of simulation facilitation^f^	.363	.466	.083	.469	.060	.471	.100	.350	.092	.523	.027	.738
Corr. R^2^	.134		.134		.073		.115		.149		.003	

^a^reference: male; ^b^reference: elderly care; ^c^ reference: advanced training; ^d^reference: no; ^e^reference: no; ^f^ continuous co-variable, bold values indicate significant results regarding 5%-level

## Discussion

It is considered that simulation in nursing education improves the quality of care in nursing because it can address gaps in the quality of education. Teaching in a skills lab prepares nurses for nursing activities, teaches nursing skills and abilities, and can influence the level of reflection of students [24]. A prerequisite is that facilitators show certain competencies regarding the INACSL standards. In the present study, against the background of the deficit of instruments to assess the competencies of simulation facilitators, the FCR was translated into German (FCR_G_) and psychometrically tested as a self-assessment instrument. This procedure could be followed for further instrument translations, so that the range of established instruments is increased, and ultimately the quality of simulation pedagogy is enhanced. Additionally, factors associated with higher competencies were evaluated. This is necessary, because Germany has only just started to carry out nursing simulation training.

Included in the study were 100 simulation teachers from universities, vocational schools, and further and continuing education in the German nursing education landscape, who completed the translated version of the FCR (FCR_G_). The participants had an average of 14.7 years of clinical nursing experience, with an average of 6.9 years since their last activity in direct patient care.

It should be noted that good simulation teachers can create a high degree of realism in scenarios if they can incorporate experiential knowledge, that is, if they can draw on a repertoire of real examples from nursing care [25, 26]. Future projects should evaluate accurate clinical experience factors (e.g., period of clinical activity, experience in different settings, past period without clinical activity besides simulation teaching) and their association with the factors affecting the outcome of simulation experience.

The self-administered FCR_G_ showed excellent reliability in terms of internal consistency and intra-rater reliability. Compared to the inter-rater reliability of the original version (ICC ≥ .77), the intra-rater reliability of the German version is high. The validity testing showed a good convergent validity due to the moderate positive correlation between FCR_G_ total score and motivation. Additionally, the structure of the FCR_G_ was confirmed by the CFA. The FCR_G_ can be used to measure facilitators’ competencies in nursing simulations in Germany, which is an important step in improving the quality of simulations and overcoming the gap with international standards. This is important because in Germany there is a lack of generally accepted standards for nursing simulation. In a future step, the agreement between self- and proxy-rated competencies using the FCR_G_ should be evaluated.

Policard notes that the complex activity of facilitating in simulations requires the ability to deal with a variety of demands [7]. Here, a gap in the specific training of facilitators is identified. Only 17% of the participants had had basic training. This might be due to the short history of simulation pedagogy in nursing in Germany. However, this number should be increased, since basic training is associated with higher competencies.

In the present study, facilitators with basic training in simulation pedagogy showed higher values of competencies for the FCR_G_ total score and pre- and debriefing domains. This emphasizes the statement of the INACSL *Simulation Facilitation* standard [10] that facilitators need basic training. Such training would improve their competencies and therefore improve the quality of simulation training. No association was found with the number of years of working experience, although it could have been assumed that longer work experience would lead to higher competencies. The analysis yielded a low proportion of explained variance. In future studies, further potential associated variables should be considered.

### Limitations

The present study used pragmatic sampling. A response rate could not be calculated since it is unknown whether the people contacted forwarded the e-mail to other facilitators. Although a comprehensive recruitment strategy was applied, a sample bias cannot be excluded. Because of the small number of participants, the results should only be generalized with care. Furthermore, the low number of participants with basic training might have an influence on the FCR_G_ ratings. It might be that people rated their competencies without respect to the INACSL background.

## Conclusions

The present study shows that the FCR-German version [27] is a suitable self-assessment tool for the evaluation of the competencies of facilitators in nursing simulation. The results imply that the FCR_G_ should be used to assess facilitators’ competencies on a regular basis. This would allow them to develop further competencies that are needed to facilitate nursing simulations. This is necessary to bring German nursing simulation to an international standard such as that recommended by the INACSL [10].

An in-depth study to identify the factors associated with higher competencies should be conducted. The FCR can, for the first time, be used to promote the international exchange of experience and knowledge of teachers in simulation.

## Data Availability

The datasets generated and/or analysed during the current study are not publicly available because of ethical concerns, but are available from the corresponding author on reasonable request.

## References

[1] Lee J-Y, Lee SH, Kim J-H (2018). A review of the curriculum development process of simulation-based educational intervention studies in Korea. Nurs Educ Today..

[2] Kirsten A, Kagermann D. Simulation in der Berufsbildung der Pflege [Simulation in nursing education]. In: St.Pierre M, Breuer G, editors. Simulation in der Medizin [Simulation in medicine]. Berlin: Springer; 2018. 447-468.

[3] Gaba DM (2004). The future vision of simulation in health care. Qual Saf Health Care..

[4] Kim J, Park JH, Shin S (2016). Effectiveness of simulation-based nursing education depending on fidelity: a meta-analysis. BMC Med Educ..

[5] Hayden JK, Smiley RA, Alexander M, Kardong-Edgren S, Jeffries PR. The NCSBN national simulation study: a longitudinal, randomized, controlled study replacing clinical hours with simulation in prelicensure nursing education. J Nurs Regul. 2014;5(2, Supplement):S3-S40. 10.1016/S2155-8256(15)30062-4.

[6] Daniels AL. Clinical simulation in pre-licensure nursing students: improving learning outcomes in psychologically safe learning environments. 2018. PhD dissertation, University of Maryland Nursing School.

[7] Policard F (2018). Facilitation and clinical simulation: modalities of guidance by nurse trainers in simulation exercise. Activites..

[8] Johnston S, Tutticci N, Theobald K, Ramsbotham J. Comparison of simulation observer tools on engagement and maximising learning: a pilot study. Int. J Nurs Educ Scholarsh. 2021;18(1):10.1515/ijnes-2019-0110. 10.1515/ijnes-2019-0110 33735945

[9] Hull L, Russ S, Ahmed M, Sevdalis N, Birnbach DJ (2017). Quality of interdisciplinary postsimulation debriefing: 360 degrees evaluation. BMJ Simul Technol Enhanc Learn..

[10] INACSL. INACSL Standards of best practice: simulation facilitation. Clin Simul Nurs. 2016;12:S16-S20. 10.1016/j.ecns.2016.09.007.

[11] Mulder M, Gulikers J. The SAGE Handbook of Workplace Learning. SAGE Publications Ltd; 2011. https://sk.sagepub.com/reference/hdbk_workplacelearning.

[12] Morse CJ, Fey M, Kardong-Edgren S, Mullen A, Barlow M, Barwick S. The changing landscape of simulation-based education. A review of the use of simulation in nursing education, professional development, and beyond. AJN. 2019;119(8):42-48. 10.1097/01.NAJ.0000577436.23986.81 31356329

[13] Frandsen A, Lehn-Christiansen S. Into the black-box of learning in simulation debriefing: a qualitative research study. Nurse Educ Today. 2020;88:104373. 10.1016/j.nedt.2020.104373. 10.1016/j.nedt.2020.104373 32145475

[14] Forstrønen A, Johnsgaard T, Brattebø G, Reime MH. Developing facilitator competence in scenario-based medical simulation: presentation and evaluation of a train the trainer course in Bergen, Norway. Nurse Educ Prac. 2020;47:102840. 10.1016/j.nepr.2020.102840. 10.1016/j.nepr.2020.102840 32745955

[15] Kilroy S, Kent D, VanderZwan KJ (2021). Development of a multisite nursing simulation work group focusing on the international nursing association for clinical simulation and learning standards. J Nurs Educ..

[16] Leighton K, Mudra V, Gilbert GE (2018). Development and psychometric evaluation of the facilitator competency rubric. Nurs Educ Perspect..

[17] IBM Corp. Released. IBM SPSS Statistics for Windows, Version 28.0. 2021.

[18] van Griethuijsen RALF, van Eijck MW, Haste H (2015). Global patterns in students’ views of science and interest in science. Res Sci Educ..

[19] Koo TK, Li MY (2016). A guideline of selecting and reporting intraclass correlation coefficients for reliability research. J Chiropr Med..

[20] The Core Team. The R Project for Statistical Computing. Accessed 07.02.2023, https://www.r-project.org/.

[21] Rosseel Y. lavaan: An R Package for structural equation modeling. J Stat Software. 2012;48(2):1 - 36. 10.18637/jss.v048.i02.

[22] Hu Lt, Bentler PM. Cutoff criteria for fit indexes in covariance structure analysis: conventional criteria versus new alternatives. Struct Equation Model. 1999;6(1):1-55. 10.1080/10705519909540118.

[23] Matsunaga M (2008). Item parceling in structural equation modeling: a primer. Commun Methods Measures..

[24] Husebø SE, O'Regan S, Nestel D (2015). Reflective practice and its role in simulation. Clin Simul Nursing..

[25] Arthur C, Kable A, Levett-Jones T (2011). Human patient simulation manikins and information communication technology use in Australian schools of nursing: a cross-sectional survey. Clin Simul Nurs..

[26] Keskitalo T (2011). Teachers’ conceptions and their approaches to teaching in virtual reality and simulation-based learning environments. Teach Teach..

[27] Forbrig TA. Translation of the facilitator competency rubric into German. https://sites.google.com/view/evaluatinghealthcaresimulation/fcr/fcr-german-version. Accessed 06.02.2023.

